# Functional seizures: Are they consistent over time?

**DOI:** 10.1002/brb3.3375

**Published:** 2024-01-29

**Authors:** Elian Bahous, Raz Wagner, Moshe Herskovitz

**Affiliations:** ^1^ Department of Neurology Rambam Health Care Campus Haifa Israel; ^2^ Technion Institute of Technology Rappaport Faculty of Medicine Haifa Israel

**Keywords:** consistency, functional seizures, stereotypy

## Abstract

**Background:**

Our previous study showed that functional seizures (FS) are consistent in the same patient during a single video EEG monitoring (VEEG). This study aimed to check whether FS remains consistent across VEEG sessions even after several years.

**Methods:**

The study evaluated the consistency of FS across different VEEG sessions using five criteria: FS type, the main anatomical region involved (specifically, the body part most affected during the seizure), other involved anatomical regions, frequency of movements, and duration of FS. Consistency levels were categorized as low (one consistent axis), moderate (two consistent axes), and high (three or more consistent axes).

**Results:**

Fourteen patients were included in the final analysis. The mean time between monitoring was 3.8 ± 2.5 years (0.5–8 year). In 13 of 14 patients, the first and second monitoring events were classified into the same FS category. There was consistency in the main anatomical region involved in 9 out of 12 patients with motor FS. In 9 out of 12 patients with motor FS, the other anatomical regions involved were consistent in both sessions. The mean duration of the FS between sessions was inconsistent in most of the patients. Ten patients were classified with high consistency, one patient with moderate consistency, two patients with low consistency, and in one patient, the events were classified as inconsistent.

**Conclusions:**

Our results show that FS tends to remain consistent in a single patient even after several years, and there is probably no correlation between the degree of consistency and the time between VEEG sessions. These findings have implications for supporting the concept of FS as a consistent phenomenon. Additionally, they may suggest potential avenues for future research to elucidate the origins of FS. Subsequent studies are essential to validate and expand upon these preliminary observations.

## 1 BACKGROUND

Functional seizures (FS) are paroxysmal events resembling epileptic seizures but lack characteristic concomitant epileptic activity, likely stemming from a range of biopsychosocial risk and perpetuating factors (Lafrance et al., 2013). The pathophysiology of FS remains unclear, with various hypotheses proposed over the years (Bautista et al., 2008; Brown & Reuber, 2016b; Reuber, 2009; Schneider et al., 2022).

The integrative cognitive model (ICM) offers a comprehensive explanation, suggesting FS represents an automatic, uncontrollable behavior akin to a conditioned reflex arising from distorted memory (Brown & Reuber, 2016b). The “seizure scaffold,” or behavior during the seizure, may originate from inherent reflexes (e.g., freeze and startle), physical symptoms (e.g., pre‐syncope, dissociation, hyperventilation, and head injury), personal knowledge, or modeling. Similar to a conditioned reflex, the seizure can be triggered by external or internal stimuli.

In a prior study (Schneider et al., 2022), we observed a “preictal” phase of inactivity in up to 70% of FS patients with motor FS, indicating that this phase is integral to FS itself. We suggested FS could represent a reactivation of a “freeze discharge” reaction, a state of immobility animals use to evade potential predators, followed by a seizure‐like phase after awakening.

Our model aligns with the ICM but is more specific. Both conceptual models depict a reflex triggered by external or internal stimuli. Although the ICM posits a conditioned reflex, our “freeze discharge” model emphasizes a reactivation of an inherent reflex. Consistent stereotypic patterns observed in previous studies support the hypothesis that FS resembles a conditioned/inherent reflex executed without high‐level processing (Asadi‐Pooya, 2019; Asadi‐Pooya et al., 2017; Herskovitz, 2017; Hubsch et al., 2011; Seneviratne et al., 2010; Vogrig et al., 2019). Our current study examined FS semiology over time in patients with separated video EEG monitoring (VEEG), hypothesizing that, in line with both models, FS semiology should remain consistent over the years.

## 2 METHODS

This study is an observational retrospective study. We retrospectively reviewed medical records and VEEG reports of all patients with FS diagnosis who underwent VEEG at our center from July 29, 2007 to November 24, 2020.

We included only patients who had at least two different VEEG sessions and had at least one recorded FS in each session.

Medical details, including age, sex, medical background, antiseizure medications, time from the beginning of events to admission to the first VEEG session, were extracted from the patients’ medical files.

We perform VEEG using a 22‐channel digital VEEG system with electrodes arranged according to a modified 10–20 system.

An epileptologist diagnosed FS through the observation of the absence of electrographic paroxysmal changes occurring during, after, or before a typical event, excluding potential causes from other epileptic imitators, such as syncope, hypoglycemia, movement disorders, and similar conditions (Lafrance et al., 2013). All FS occurred spontaneously. It is important to note that during VEEG, we check with the patient and his family members that the events occurring during VEEG are the typical events occurring at home.

This study is a modification of our previous study in which we checked consistency in patients who had more than one FS during a single VEEG, due to different inclusion criteria patients in the current study are not shared with patients in the previous study.

The consistency of FS between sessions was evaluated using five axes: 1. Type of FS: (1) rhythmic motor FS characterized by rhythmic tremor or rigor‐like movements; (2) hypermotor FS characterized by violent movements; (3) complex motor FS characterized by complex movements such as flexion, extension, abduction, adduction, rotation, with or without clonic and myoclonic‐like components of varying combinations and anatomic distribution; (4) dialeptic FS is characterized by unresponsiveness without motor manifestation; (5) non‐epileptic aura characterized by subjective sensations without any external manifestations; (6) mixed FS with combinations of the above seizure types (adopted from Seneviratne et al. (2010)). Patients with nonepileptic aura characterized by subjective sensations without any external manifestations were excluded from this study. (1) The main antomical region involoved (MARI). (In our previous study, we found that in all motor FS, there was a main anatomical region involved during the seizure which we named—MARI (Herskovitz, 2017).) (2) Other anatomical regions involved during FS. (3) Frequency of MARI movements calculated using the EEG movement artifact. In case of no movement artifact, we estimated the frequency using frame‐by‐frame video analysis: 1/ duration of one movement cycle. (4) Duration of FS.

For each patient, we analyzed the first event in each VEEG session. One patient had a second event during a short‐term EEG and in one patient the first event during VEEG was compared to a follow‐up event captured by a home camera. If we were unable to retrieve video footage of an event, we analyzed the event according to the description in the VEEG report. In our center, we use a structured VEEG report which includes a detailed description of the seizure, from which one can easily extract the type of FS, main anatomical region involved, accompanying anatomical regions involved, and duration of events.

We defined three levels of consistency: (1) Low consistency–consistency between events was observed in one axis. (2) Moderate consistency–consistency was observed in two axes. (3) High consistency–consistency was observed in three axes or more.

In a case where none of the axes was consistent, the events were regarded as non‐consistent.

We used descriptive statistics to analyze axes 1–3. We conducted two‐tailed *t*‐tests to compare MARI's mean movement frequency and the mean duration of FS events during different VEEG sessions. Additionally, we computed the Δ in MARI's movement frequencies and considered a Δ less than 10% as indicative of consistency. Similarly, for seizures’ duration, we calculated the Δ and regarded a Δ less than 20% as consistent.

We used windows SPSS v 25 for statistical analysis.

Our local institutional review board approved all study procedures.

## 3 RESULTS

Between July 29, 2007 and November 24, 2020, we retrieved 432 VEEG sessions with a diagnosis of PNES. Fourteen of them had more than one VEEG session and 12 had at least one recorded event in each admission.

A patient who was admitted due to events exacerbation had a recorded seizure during a short‐term EEG recording session, and another patient who was supposed to be readmitted for VEEG had her seizure recorded at home on video without EEG. Therefore, 14 patients were included in the final analysis.

The mean time from onset of FS events to the first VEEG was 2.19 ± 1.62 years (0.1–18 years) when excluding the patient with 18 years duration of FS events, the meantime from the onset of FS events to the first VEEG was 0.97 ± 0.8 years (0.1–2 years).

In six patients, we were able to compare events using actual video analysis, while in three patients, we had a video recording in only one of the sessions, and in five patients, we had no video recording available. When no video recording was available, we compared the events relying on a detailed structured description of the events in the formal VEEG report. The mean age of the patients was 41 ± 17 (16–72) years, there were seven men and seven women, and the mean time between monitoring sessions was 3.8 ± 2.5 (0.5–8) years.

Table 1 summarizes the data of the patients.

**TABLE 1:** “Patients” characteristics.

Mean age (years)	41 ± 17
Meantime between VEEG (years)	3.8 ± 2.5
Men	7 (50%)
Women	7 (50%)
Interictal epileptiform discharges	4 (28%)
Psychiatric comorbidity	5 (35%)
ASM prescription	10 (71%)

Abbreviations: ASM, anti‐seizure medications; VEEG, video‐EEG monitoring.

Reasons for readmission included an increase in frequency of events in four patients, suspected epileptic seizures in seven patients, exacerbation of the intensity of the event in one patient, and unknown reasons in two patients.

All patients reported the events as their habitual seizures.

In 13 of 14 patients, the first and second events were classified in the same FS category: 7 rhythmic motor, 1 mixed FS (dialeptic + rhythmic motor), 3 complex motor, and 2 dialeptic in both sessions.

In one patient, the event in the first VEEG session was of a rhythmic motor type, and in the second session, the patient suffered a panic attack.

**FIGURE 1:**
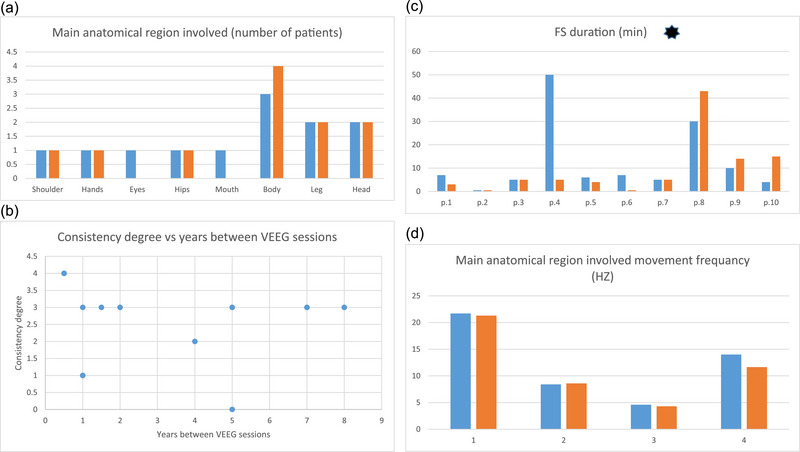
Results summary. 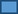
—First session. 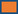
—Second session. 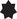
—mean functional seizures (FS) duration 12.4 ± 15.4 versus 9.5 ± 12.7 *p* = .031. VEEG, video EEG monitoring. (a) shows the main anatomical region involved in both sessions; (b) shows the consistent degree vs years between VEEG sessions. Every dot represents a patient, the x‐axis represents years between sessions, and the y‐axis represents a consistent degree; (c) shows a comparison of FS duration between sessions; (d) shows comparison of MARI movement frequency in 4 patients between sessions.

Graph 1 summarizes the main results regarding the FS characteristics in both VEEG sessions. In 9 out of 12 patients with motor manifestations, there was consistency in MARI. In two patients (p.5 and p.6), there was a change in MARI without a change in the FS category and in one patient (p.11), there was a change in category (Figure 1a).

In 9 out of 12 patients with motor FS, the other anatomical regions involved were consistent in both sessions (Table 2).

**TABLE 2:** Anatomical regions involved in each session and sequence of their appearance.

Patient no.	FS 1	FS 2
1	Head—nonresponsive	Head—nonresponsive
2	Right leg	Right leg
3	Whole‐body	Whole‐body
4	Whole‐body	Whole‐body
5	Mouth—right hand and leg—left leg	4 limbs
6	Right hand and leg‐left hand—4 limbs	Both hands—4 limbs
7	Face and head	Face and head
8	Eyes—nonresponsive	Left hand—nonresponsive
9	Hands—nonresponsive‐whole body	Hands—nonresponsive‐whole body
10	Dialeptic	Dialeptic
11	Right hand—face and back—Hip—legs	Panic like
12	Dialeptic	Dialeptic
13	Legs and hip	Legs and hip
14	Shoulders—legs (mainly left)	Shoulders—whole body

Abbreviation: FS, functional seizures.

In 10 patients, the duration of the events was available in both VEEG sessions. In 7/10 patients, the duration between sessions was larger than 20%. The mean duration of FS between VEEG sessions was significantly different: 12.4 ± 15.4 versus 9.5 ± 12.7 *p* = .031 (Figure 1c). The MARI movement frequency remained consistent in three of four patients with motor FS and available video recordings from both sessions (P1 Δ = 2%, P2 Δ = 2.4%, P3 Δ = 6.6%, and P4 Δ = 17%) (Figure 1d).

Ten patients were classified with high consistency, one patient with moderate consistency, two patients with low consistency, and in one patient, the events were classified as inconsistent. No correlation was found between the degree of consistency and the time between sessions (Figure 1b).

## 4 DISCUSSION

This study was conducted to examine whether FS are consistent over time for the same patient. Thirteen out of 14 patients had seizures with concordant semiology classifications in both sessions. Nine out of 12 patients with motor FS had the same MARI in both sessions. In 9 out of 12 patients with motor FS, the same anatomical regions were involved (two patients had dialeptic FS in both VEEG sessions). Three out of four patients had similar movement frequencies during both sessions. A total of 10 patients were classified as highly consistent, 1 as moderately consistent, 2 as lowly consistent, and 1 as inconsistent. Additionally, no correlation was found between consistency and the time between sessions.

The categorization of FS and the measurement of their consistency remain controversial (Duwicquet et al., 2017). It was Seneviratne et al. (2010) who first suggested the stereotypicity of FS semiology within the same patient. Their study addressed the semiological classification of FS, delineating it into six distinct types. However, they found that 82% of cases of FS existed within a single semiological category.

In a prior study, we explored the semiological consistency across events within the same VEEG session using five axes of analysis (Bautista et al., 2008). (1) Type of FS, (2) Main anatomical region involved during FS, (3) Other anatomical regions involved during the FS, (4) Frequency of movement, and (5) Duration of the events (Herskovitz, 2017). Our findings revealed consistent classification within the same category for the first two events across all patients. Moreover, there was a persistent involvement of the main anatomical region (MARI) in all motor FS events, and for 13 out of 14 patients with motor FS; there was concordance in the main antomical region involoved (MARI) involvement, coupled with consistency in the frequency of movement. However, it is noteworthy that there was inconsistency observed in the duration of the events (Herskovitz, 2017).

Moreover, several studies, employing varied methodologies (Asadi‐Pooya, 2019; Reuber, 2009), have indicated the consistency of FS. Vogrig et al. (2019) utilized a behavioral mapping approach, evaluating consistency across four categories: duration, sequence, type, and continuity. Their study demonstrated comparable consistency between FS and temporal lobe seizures.

In our perspective, the current study, presenting supplementary evidence affirming the consistency of FS, not only in the analysis of similarities between two attacks occurring in close temporal proximity but also those separated by a significant interval, may contribute to advancing our comprehension of their etiological origins.

Various models have offered diverse interpretations of FS, ranging from the activation of dissociated material to physical manifestations of emotional distress, hard‐wired reflex responses, or learned behaviors. However, most of these models fall short in providing a comprehensive explanation for the phenomenology of FS (Almis et al., 2013; Bakvis et al., 2009; Binder et al., 1994; Bogaerts et al., 2010; Cassady & Baslet, 2023; Huepe‐Artigas et al., 2022; Lanius et al., 2012).

In contrast, the ICM, proposed by Reuber and Brown, puts forward a more encompassing framework (Brown & Reuber, 2016a, 2016b; Reuber, 2009). According to the ICM, the observable and subjective aspects of FS arise from the automatic execution of a learned mental representation referred to as the “seizure scaffold.” This scaffold comprises a sequence of perceptions and motor activities shaped by experiences like inherent reflexes, physical symptoms, personal knowledge, or modeling. The automatic execution of this scaffold is theorized to be triggered by internal or external stimuli, often associated with heightened autonomic arousal. It results in the disruption of awareness of distressing material and is subjectively experienced as non‐volitional.

In a previous study (Schneider et al., 2022), we proposed that FS might indicate a reactivation of a “freeze discharge” reaction—a state of immobility employed by animals to evade potential predators—followed by a seizure‐like phase upon awakening. This conceptual framework aligns with the ICM, offering greater specificity by elucidating the probable underlying mechanisms in only a subset of patients with FS (70%). This model is also consistent with other models suggesting that FS represent activation of primitive nonconscious defensive behaviors (such as that driven by subcortical structures including the periaqueductal gray) (Jungilligens et al., 2022). Nevertheless, the observed phenomenon is interpreted as a reflex in both models. Although Reuber (2009) viewed FS as a conditioned reflex, our model interprets FS as a reactivation of an inherent reflex. Regardless of whether the reflex is conditioned or inherent, its activation occurs without the involvement of higher processing levels, and in most cases, a similar activation pattern is anticipated (Brown & Reuber, 2016a, 2016b; Jungilligens et al., 2022; Reuber, 2009).

In the aforementioned study conducted by Herskovitz (2017), a restricted cohort of patients with multiple recorded FS during monitoring exhibited stereotypic features. Notably, patients experiencing either motor rhythmic or complex motor seizures consistently manifested involvement of a primary anatomical region, indicative of a uniform seizure scaffold. The researchers posited that this observation aligns with the hypothesis of a dissociative state.

Defining the timing and mechanisms underlying the generation of the seizure scaffold proves even more challenging. However, by considering three key observations: (1) the high prevalence of emotional trauma and sexual abuse history in FS patients; (2) the non‐volitional and automatic nature of execution, often triggered in circumstances associated with heightened autonomic arousal; and (3) a greater likelihood of activation with increased dysfunctional inhibition (Brown & Reuber, 2016b). Our recent findings, albeit with limited statistical support, indicating the gross stability of the seizure scaffold over time raise a hypothesis. It is plausible that this enduring, non‐volitional, unconscious, stress‐related seizure scaffold may have originated in correlation with the initial emotional injury, occurring either during the traumatic event itself or in subsequent attempts to evade the repercussions of the painful memory.

To examine this hypothesis in future research, a detailed analysis of cases with prior diagnoses of FS linked to psychological trauma is warranted. This involves a meticulous review of instances with detailed traumatic event descriptions, followed by a comparative analysis with associated seizure scaffolds. Concerns about patient privacy and well‐being and potential lack of documented, detailed questioning may pose significant obstacles to pursuing this research avenue.

Finally, our study is constrained by a small sample size, a retrospective design, limited availability of complete video footage for the first and second sessions (observed in only six patients), patients were only queried about habitual seizures, not others, and the lack of real‐time accelerometers for frequency measurement—relying instead on EEG movement artifact or frame‐by‐frame video analysis for calculating the Main antomical region involoved (MARI) movement frequency. Furthermore, our study included only 14 patients out of 432 individuals with FS studied at our center, which can lead to a substantial selection bias, in which the patients in our study do not represent the entire FS population but rather represent patients with more prolonged and refractory condition.

## 5 CONCLUSIONS

Significantly, this study is the first, to our knowledge, to explore the semiological consistency of FS over time. Our results show that FS tends to remain consistent in a single patient even after several years, and there is probably no correlation between the degree of consistency and the time between VEEG sessions. Despite its limitations, these findings have implications for supporting the concept of FS as a consistent phenomenon. Additionally, they may suggest potential avenues for future research to elucidate the origins of FS. Subsequent studies are essential to validate and expand upon these preliminary observations.

## AUTHOR CONTRIBUTIONS

**Elian Bahous**: Investigation; writing—original draft. **Raz Wagner**: Validation; writing—review and editing. **Moshe Herskovitz**: Conceptualization; supervision; methodology; writing—review and editing.

## CONFLICT OF INTEREST STATEMENT

The authors have no disclosures or conflicts of interest to report.

## FUNDING INFORMATION

No funding was needed for the study.

### PEER REVIEW

The peer review history for this article is available at https://publons.com/publon/10.1002/brb3.3375.

## Data Availability

The data that support the findings of this study are available on request from the corresponding author. The data are not publicly available due to privacy or ethical restrictions.
